# Summary of the National Advisory Committee on Immunization (NACI) Updated Guidance on Influenza Vaccination During Pregnancy

**DOI:** 10.14745/ccdr.v50i34a01

**Published:** 2024-04-30

**Authors:** Winnie Siu, Angela Sinilaite, Jesse Papenburg

**Affiliations:** 1 Centre for Immunization Programs, Public Health Agency of Canada, Ottawa, ON; 2 School of Epidemiology and Public Health, Faculty of Medicine, University of Ottawa, Ottawa, ON; 3 NACI Influenza Working Group Chair; 4 Division of Pediatric Infectious Diseases, Department of Pediatrics, Montréal Children’s Hospital, McGill University Health Centre, Montréal, QC; 5 Division of Microbiology, Department of Clinical Laboratory Medicine, OPTILAB Montréal-McGill University Health Centre, Montréal, QC; 6 Department of Epidemiology, Biostatistics and Occupational Health, School of Population and Global Health, McGill University, Montréal, QC

**Keywords:** National Advisory Committee on Immunization, NACI, influenza, influenza vaccine, pregnancy, guidance

## Abstract

**Background:**

Seasonal influenza infection can lead to serious complications and adverse outcomes for pregnant individuals, the developing fetus and infants younger than six months of age. This supplemental statement provides an evidence summary on the safety and effectiveness of influenza vaccination in pregnant individuals, and the benefits and risks to the pregnant person, the developing fetus and infants younger than six months of age.

**Methods:**

A systematic review was conducted on the effectiveness and safety of influenza vaccination in pregnancy. The National Advisory Committee on Immunization (NACI)’s evidence-based process was used to assess the quality of eligible studies, summarize and analyze the findings, and apply an ethics, equity, feasibility and acceptability lens to develop recommendations.

**Results:**

The evidence suggests that influenza vaccination during pregnancy is effective in reducing the risk of laboratory-confirmed influenza infection and hospitalization in both pregnant individuals and their infants up to six months postpartum. The evidence also suggests that influenza vaccination during pregnancy does not increase the risk of non-obstetric serious adverse events in pregnant persons, infant death, spontaneous abortion, stillbirth, preterm birth, small for gestational age, low birth weight and congenital anomalies.

**Conclusion:**

Based on this body of evidence, NACI reaffirms the safety and importance of influenza vaccination during pregnancy. NACI recommends that individuals at any stage of pregnancy should receive an age-appropriate inactivated, unadjuvanted or recombinant influenza vaccine each influenza season. Influenza vaccination may be given at the same time as, or at any time before or after administration of another vaccine, including the coronavirus disease 2019 (COVID-19) or pertussis vaccines.

## Introduction

Pregnant individuals are at higher risk for severe influenza disease and related complications such as pneumonia, hospitalization and death, compared to non-pregnant individuals, because of pregnancy-related changes in anatomy and the immune and cardiovascular systems ((1-3)). Influenza infection during pregnancy can also impact the developing fetus and increase the risk of late-stage pregnancy loss, stillbirth, preterm birth and low birth weight ((3,4)). Furthermore, infants younger than six months of age are also at high risk for severe influenza disease and complications but are too young to be eligible for influenza vaccination; however, passive transfer of antibodies from influenza vaccination during pregnancy can protect newborns during their first months of life. Therefore, the National Advisory Committee on Immunization (NACI) has identified pregnant individuals as a high-risk group for whom influenza immunization is particularly important, and strongly recommends immunizing pregnant persons against influenza to protect both them and their infants from severe disease. Despite pregnant people being prioritized to receive influenza vaccine, uptake remains lower than the non-pregnant population.

Literature continues to be published on influenza vaccination in pregnancy and NACI has taken this opportunity to review the safety, efficacy and effectiveness of influenza vaccines in pregnancy. NACI’s *Updated Guidance on Influenza Vaccination During Pregnancy* statement ((5)) aims to synthesize key information and evidence to support provincial and territorial vaccine programs and frontline vaccinators in offering influenza vaccine to pregnant individuals. The statement supplements NACI’s overarching recommendations for influenza vaccination, which are available in the NACI *Statement on Seasonal Influenza Vaccine for 2023–2024* ((6)).

## Methods

The policy question addressed in this statement is: Should pregnant individuals, at any stage of pregnancy, continue to be listed among those who are particularly recommended to receive influenza vaccination? To address this question, a *de novo* systematic review was conducted to gather evidence to inform NACI’s recommendations regarding the use of influenza vaccines during pregnancy. The methodology was specified *a priori* in a published protocol ((7)). For a comprehensive description of the review methods, including details on the study eligibility, literature search, study selection, data collection and statistical methods, please refer to Wolfe *et al.* ((7)). The review protocol and knowledge synthesis were developed and performed in collaboration with the Methods and Applications Group for Indirect Comparisons through the Drug Safety and Effectiveness Network (DSEN) and supervised by the NACI Influenza Working Group. An update to the literature search was completed by the NACI Secretariat in conjunction with a librarian from the Health Library of Health Canada and the Public Health Agency of Canada (PHAC). Methods related to the review update completed by the NACI Secretariat are reported in Appendix A of the supplemental statement. A health economic analysis was not conducted as it was not deemed necessary for this statement. In addition to critically appraising evidence on burden of disease and vaccine characteristics such as safety, efficacy, immunogenicity and effectiveness, NACI applied the Ethics, Equity, Feasibility, and Acceptability (EEFA) Framework with accompanying evidence-informed tools (Ethics Integrated Filters, Equity Matrix, Feasibility Matrix, Acceptability Matrix) to systematically consider these programmatic factors for the development recommendations ((8)). The NACI evidence-based process was used to assess the available evidence and develop updated recommendations.

## Results

### Vaccine efficacy/effectiveness

The DSEN systematic review assessed the effect of seasonal influenza vaccination during pregnancy against influenza-related infection and hospitalization in pregnant persons and/or their infants using findings from four randomized controlled trials (RCTs) ((9-12)) and two observational studies ((13,14)). Additional observational studies (n=6) were identified from the updated literature search, reporting data on influenza vaccine effectiveness in pregnant persons and/or their infants up to six months of age ((15-20)).

### Benefits to the pregnant person: Vaccine efficacy/effectiveness

Overall, four studies reported data on laboratory-confirmed influenza (LCI) infection and three reported data on hospitalization due to LCI infection during pregnancy or up to six months post-partum.

A meta-analysis of the three RCTs suggested that seasonal influenza vaccination during pregnancy reduces the risk of LCI infection in pregnant persons prior to delivery and up to six months postpartum (pooled vaccine effectiveness [VE]=50%; 95% CI: 22–68, I^2^=49%). One prospective cohort study conducted during the 2019–2020 influenza season in Greece also found a protective effect of seasonal IIV4 (quadrivalent inactivated influenza vaccine, IIV) against LCI infection in pregnant persons (adjusted vaccine effectiveness [aVE]=44%; 95% CI: 28–56) ((15)).

A meta-analysis of two test-negative studies suggested that seasonal influenza vaccination during pregnancy reduces the risk of hospitalization due to lab-confirmed influenza in pregnant persons prior to delivery and up to 42 days postpartum (pooled aVE=42%; 95% CI: 19–58, I^2^=0%) ((13,16)).

One prospective cohort study reported vaccine effectiveness of 38% (95% CI: 14–55) against LCI hospitalization during pregnancy or up to two days after delivery ((17)).

### Benefits to the infant: Vaccine efficacy/effectiveness

Overall, seven studies reported data on the effectiveness of influenza vaccination during pregnancy on infant LCI infection and five studies reported data on hospitalization due to LCI infection in infants up to six months of age.

A meta-analysis of the four RCTs demonstrated a protective effect of seasonal influenza vaccination during pregnancy against LCI in infants up to six months of age (pooled VE=37%; 95% CI: 22–49, I^2^=0%) ((9,11,12,21)). Results from the RCTs suggest that the greatest effect of seasonal influenza vaccination during pregnancy against LCI infection in infants was found from birth up to two months of age (pooled VE_0 to <2 months_=61%; 95% CI: 17–81, I^2^=40%), following which the protective effect of vaccination during pregnancy waned as infant age increased (pooled VE_2 to <4 months_=42%; 95% CI: −13–70, I^2^=60%, and pooled VE_4 to <6 months_=24%; 95% CI: −3–44, I^2^=0%).

A meta-analysis of the three cohort studies demonstrated a protective effect of seasonal influenza vaccination during pregnancy against LCI infection in infants up to six months of age (pooled aVE=41%; 95% CI: 23–55, I^2^=17%) ((15,18,19)).

A meta-analysis of the three test-negative studies demonstrated a protective effect of seasonal influenza vaccination during pregnancy against hospitalization due to LCI infection in infants up to six months of age (pooled aVE=42%; 95% CI: 16–59, I^2^=71%) ((14,16,20)). Two cohort studies reported data on hospitalization due to LCI infection in infants up to six months of age, but only one demonstrated a significant protective effect of influenza vaccination during pregnancy (aVE=62%; 95% CI: 9–84 ((18)), and 21%; 95% CI: −18–47 ((19))).

### Vaccine safety

The DSEN systematic review on the safety of influenza vaccination during pregnancy evaluated non-obstetric serious adverse events (AE) in pregnant persons related to the administration of seasonal influenza vaccination during pregnancy using findings from three RCTs and three cohort studies. Additionally, the systematic review included four RCTs and 24 observational studies, including 20 cohort and four case-control studies, addressing other safety and/or pregnancy/birth related outcomes (i.e., infant death, spontaneous abortion [SAB], stillbirth, preterm birth, small for gestational age, low birth weight and congenital anomalies). Eleven additional observational studies were identified from the updated literature search.

### Harms to the pregnant person

Two RCTs evaluated the risk of severe systemic reactions within seven days of seasonal influenza vaccination in pregnant people. No significant difference in the frequency of severe systemic reactions within seven days of seasonal influenza vaccination was observed within each individual study (RR 1.35; 95% CI: 0.78–2.34 ((10)), and RR 4.95; 95% CI: 0.24–102.95 ((11))). The studies found either no difference in the occurrence of serious non-obstetric AEs, no AEs related to vaccination or no serious AEs. Finally, one cohort study and one case-series reported data on Guillain-Barré syndrome (GBS) following seasonal influenza vaccination during pregnancy. The cohort study identified no cases of GBS within 42 days of intervention in 75,906 vaccinated pregnant persons and one case in 147,992 unvaccinated pregnant persons in the United States (RR 0.65; 95% CI: 0.03–15.95) ((22)). The case series identified from the updated literature search reported one case (n=239) of GBS that occurred five days after IIV4 administration during the third trimester of pregnancy in a 29-year-old woman. The woman gave birth to a healthy baby while recovering and has fully recovered ((23)).

### Harms to the infant

Four RCTs compared the effect of seasonal influenza vaccination to placebo (n=2) ((9,10)) or active comparators (n=2; meningococcal quadrivalent vaccine ((11)) or 23-valent pneumococcal vaccine ((12))) during pregnancy on infant death up to six months of age. All RCTs were conducted in low-to-middle-income countries, and the control group infant death risk ranged from 1.1% to 2.8%. A meta-analysis of these RCTs did not demonstrate an association between seasonal influenza vaccination during pregnancy and infant death (pooled RR 1.14; 95% CI: 0.86–1.50, I^2^=9%). Furthermore, no infant death was reported from a prospective cohort study conducted in Japan among infants diagnosed with fever from zero to six months of age born from vaccinated and unvaccinated pregnant people (n=0/36 IIV and n=0/47 unvaccinated) ((24)).

Three cohort studies and three observational studies evaluated the effect of IIV during pregnancy on SAB at less than 20 and 22 weeks gestational age. Two prospective cohort studies were included in a meta-analysis and no association between IIV and SAB was demonstrated (pooled aHR 0.77; 95% CI: 0.31–1.89, I^2^=38%) ((25,26)). A third prospective cohort study found the same risk of SAB at less than 22 gestational weeks (0.4%) among unvaccinated and vaccinated pregnant people (first-trimester vaccination) ((27)).

Two retrospective case-control studies conducted by the same set of investigators in the United States assessed the association between SAB and vaccination within 28 days prior to SAB. The first study was conducted over two consecutive influenza seasons following the 2009 H1N1 pandemic ((28)). The authors observed an increased risk of SAB following IIV only in the first post-pandemic season (2010–2011 aOR 3.70; 95% CI: 1.40–9.40) but not the second (2011–2012 aOR 1.40; 95% CI: 0.60–3.30). *Post hoc* analyses of 2010–2011 data found that people who had been previously vaccinated in the 2009–2010 season with the H1N1 pandemic vaccine were at increased risk of SAB following IIV in the 2010–2011 season, which was not observed in those not vaccinated with the H1N1 pandemic vaccination in 2009–2010 but vaccination with IIV in 2010–2011.

The second study conducted over three consecutive influenza seasons (i.e., 2012–2013, 2013–2014 and 2014–2015) by Donahue *et al*. sought to confirm the association observed between SAB and history of influenza vaccination ((29)). No association was found between seasonal influenza vaccination during pregnancy and SAB within 28 days of vaccination (aOR 0.80; 95% CI: 0.60–1.10), including among people vaccinated in the previous season. The authors state that the association of prior season vaccination found in the initial study may have been a spurious result due to residual confounding or random error, or it may have been due to differences in the time periods of the two studies. One cohort study identified from the updated literature search conducted in the United States over the 2008–2009 to 2013–2014 influenza seasons did not find an association between the history of pandemic H1N1-containing influenza vaccination and SAB within 28 days of vaccination (aHR 1.19; 95% CI: 0.97–1.46) ((30)).

An additional three single-arm cohort studies ((31-33)) and one case series (23)) identified from the updated literature search reported data on SAB in persons vaccinated with IIV during pregnancy. Overall, from the three single-arm cohort studies and the case series study, no safety signals were identified among pregnant persons exposed to IIV.

No safety issues were identified regarding the administration of seasonal influenza vaccines during pregnancy, with respect to other adverse birth outcomes including stillbirth (18–22 gestational weeks or at least 500 g), preterm birth, small for gestational age birth, low birth weight and congenital anomalies identified at birth or up to six months of age. Evidence was derived from both RCTs and observational studies, including case-control studies and cohort studies.

### Ethics, equity, feasibility and acceptability considerations

There were no distinct significant ethics or equity issues identified. Recommendations that allow vaccination at all gestational stages of pregnancy would reduce feasibility barriers in vaccination programs. Low vaccine uptake in pregnant individuals has been partly attributed to vaccine hesitancy, which is complex and multidimensional and can be influenced by individual, logistical, cultural and sociologic factors. A recommendation from a healthcare provider is the most important factor when deciding to be vaccinated; therefore, ensuring providers are well-informed of the most recent evidence and can communicate the importance of seasonal influenza vaccination during pregnancy is important for improving vaccine uptake.

## Recommendations

1. NACI recommends that influenza vaccine should be offered to pregnant individuals. Recommended products include: IIV-SD, IIV-cc and RIV. (*Strong NACI Recommendation*)

· There has been no identified safety signal regarding the use of RIV during pregnancy, although published clinical data are limited.

· There has been no identified safety signal regarding the use of LAIV in pregnancy, although there are more data on the safety of other influenza vaccine products in pregnancy. There is also evidence that IIV has higher efficacy than LAIV in healthy adults. Note that vaccination with LAIV during pregnancy should not be considered a reason to terminate pregnancy.

· The only adjuvanted vaccine in Canada for the 2023/2024 influenza season is IIV3-Adj, which is authorized for infants 6 to 23 months (Fluad Pediatric^®^) and adults 65 years and older (Fluad^®^). There has been no identified safety signal regarding adjuvanted influenza vaccines in pregnancy; however, IIV3-Adj is not authorized for people of reproductive age.

· The only high-dose vaccine in Canada for the 2023/2024 influenza season is IIV4-HD (Fluzone^®^ High-Dose Quadrivalent) which is authorized for adults 65 years and older. There has been no identified safety signal regarding high-dose influenza vaccines in pregnancy, however, IIV4-HD is not authorized for people of reproductive age.

2. NACI recommends that influenza vaccination should be offered at any stage of pregnancy (i.e., in any trimester). (*Strong NACI recommendation*)

· If an individual’s pregnancy extends over two influenza seasons, that person may receive two doses of influenza vaccine (i.e., one dose in each season during the course of the pregnancy).

3. NACI recommends the inclusion of all pregnant individuals, at any stage of pregnancy, among those who are particularly recommended to receive influenza vaccination. (*Strong NACI recommendation*)

4. NACI reiterates its recommendation that influenza vaccination may be given at the same time as, or at any time before or after administration of another vaccine, including COVID-19 or pertussis vaccine. (*Strong NACI recommendation*)

· Every appropriate opportunity to immunize during pregnancy, with any immunization for which the pregnant person is eligible, should be taken.

A complete review of evidence and full NACI recommendations are published in the new NACI statement: *Updated Guidance on Influenza Vaccination During Pregnancy* ((5)).

## Conclusion

Pregnant people and their fetuses and infants are at high risk of complications from influenza. The systematic review and meta-analysis conducted for this supplemental statement examined current literature on the use of influenza vaccines during pregnancy. NACI concluded that the evidence continues to support the safety and effectiveness of influenza vaccination during pregnancy and recommends the use of either inactivated or recombinant influenza vaccines. Influenza vaccination reduces the risk of influenza and has no identified link to negative outcomes in pregnant individuals or their infants. NACI is committed to following vaccine safety information and efficacy/effectiveness data for pregnant individuals as they evolve and will update guidance as needed.
